# The Continued Importance of Promoting Exercise as Part of Oncology Care for Breast Cancer Patients∗

**DOI:** 10.1016/j.jaccao.2022.08.003

**Published:** 2022-09-20

**Authors:** Alpa V. Patel, Erika Rees-Punia

**Affiliations:** Department of Population Science, American Cancer Society, Kennesaw, Georgia, USA

**Keywords:** cardio-oncology, physical activity

**Figure:**
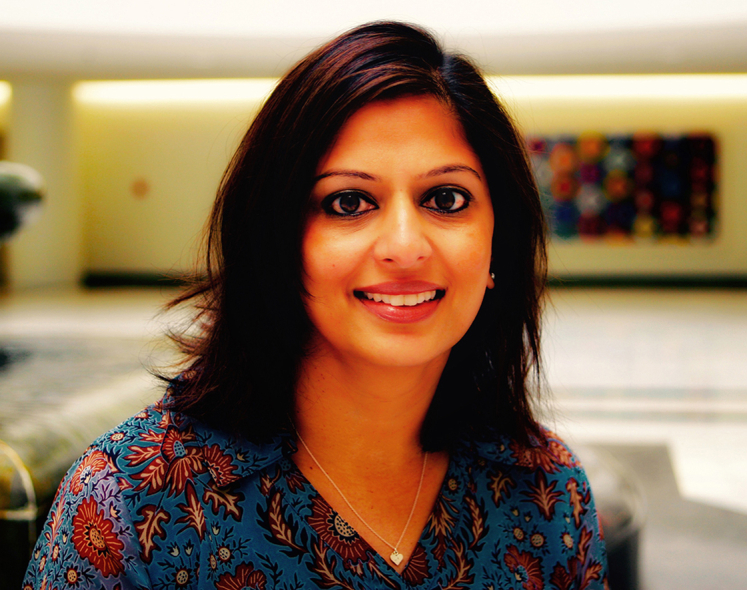

The benefits of exercise during cancer treatment are multifaceted and well-documented. Most studies demonstrate that exercise is not only safe for cancer patients undergoing treatment, but also highly beneficial for physical and mental health during and after cancer treatment.^1^, ^2^, ^3^ Exercise prescriptions are beneficial in the management of several disease- and treatment-related side effects including anxiety, depression, physical function, and lymphedema,^2^^,^^4^ maintenance of cardiac health,^5^ as well as improved overall survival, cardiovascular disease survival, and cancer-specific survival.^6^ Despite the known benefits and call to incorporate exercise during oncology treatment and care, third-party payers generally provide coverage for exercise programs to cancer patients at higher risk for cardiovascular events.

The benefits of exercise during breast cancer treatment are among the most extensively studied of any cancer type. Studies in breast and other cancer patients have demonstrated the benefits of exercise in improving cardiorespiratory fitness (CRF) in cancer patients during treatment.^7^^,^^8^ CRF often declines during treatment due to the high cardiac toxicity of many chemotherapies, particularly those used in the treatment of HER2^+^ breast cancer,^9^^,^^10^ thus strategies to mitigate any cardiac effects in cancer patients would have tremendous potential in improving longer-term outcomes including mortality.^11^ To that end, in this issue of *JACC: CardioOncology*, Peck et al^12^ examine whether self-reported moderate-to-vigorous intensity physical activity (MVPA) is associated with better quality of life, cardiac function, and post-treatment CRF in 88 women with HER2^+^ breast cancer. MVPA and outcomes were measured at 6 time points over the course of approximately 1 year: near the time of diagnosis before treatment initiation; following anthracyclines, but before initiating trastuzumab; every 3 months during trastuzumab treatment; and at the completion of trastuzumab treatment. They found that higher MVPA was associated with better quality of life, measures of cardiac function, and post-treatment CRF. The findings around cardiac function are of particular importance, given the documented negative effects of anthracyclines and trastuzumab on cardiac function for women with HER2^+^ breast cancer.^13^^,^^14^ The combination of these drugs can lead to severe cardiac damage^10^; thus, these findings offer additional evidence of the benefits of exercise in mitigating these cardiac effects specifically for HER2^+^ breast cancer patients receiving sequential anthracyclines and trastuzumab.

These findings are consistent with other prior studies of breast cancer patients and support the continued need to promote exercise as part of oncology care, but there are some limitations of this study that should be acknowledged. Many of these limitations were acknowledged by the investigators including the small sample size and use of self-reported MVPA data. The investigators stated that pre-existing cardiovascular disease risk factors contribute to poor cardiac outcomes, but they had a limited measure of important potential confounders such as prediagnosis levels of alcohol, diet quality, and excess body fatness. The investigators were also unable to explore the role of prediagnosis MVPA, which may be a limitation given the documented association between MVPA before diagnosis and improved cardiovascular disease outcomes among breast cancer survivors.^15^ Finally, the lack of activity type, strength training, and sedentary time are additional limitations. While acknowledging these limitations, it is important to note the high-quality, robust CRF measures in the present study are a major strength.

Future studies addressing these limitations will be important and should, in particular, include: 1) measures of quality of life and CRF before treatment initiation to examine treatment-related changes; and 2) more detailed measures of physical activity measured before and during treatment. Although the Godin physical activity survey instrument used in the present study has shown moderate comparability with other self-reported measures of MVPA, the lack of detail on MVPA type, strength training, and sedentary time prevent analyses on of the roles of these distinct domains. As cancer survivor-specific recommendations for physical activity include aerobic exercise, resistance training, or a combination of both for expected patient benefits, future studies relying on self-report should consider newer, more detailed, validated survey instruments that capture all of these activity domains.^16^^,^^17^

This study reinforces the benefits of MVPA during treatment for breast cancer, here particularly for HER2^+^ breast cancer patients receiving anthracyclines followed by trastuzumab. Given the well-established benefits of exercise on physical and mental health, various patient-reported outcomes, improved survival, and other endpoints among cancer survivors, and the mounting evidence for improving CRF during treatment, guidelines are increasingly calling for the incorporation of exercise into oncology care.^1^^,^^3^^,^^18^ Most studies support that exercise is generally safe for individuals undergoing cancer treatment. Oncology care teams should encourage breast cancer patients to incorporate exercise as part of their treatment plan.

## Funding Support and Author Disclosures

The authors have reported that they have no relationships relevant to the contents of this paper to disclose.
